# Modeling, Fabrication and Testing of a Customizable Micromachined Hotplate for Sensor Applications

**DOI:** 10.3390/s17010062

**Published:** 2016-12-30

**Authors:** Alessio Tommasi, Matteo Cocuzza, Denis Perrone, Candido Fabrizio Pirri, Roberto Mosca, Marco Villani, Nicola Delmonte, Andrea Zappettini, Davide Calestani, Simone Luigi Marasso

**Affiliations:** 1 χlab—Materials and Microsystems Laboratory, Department of Applied Science and Technology, Politecnico di Torino—Via Lungo Piazza d’Armi 6, 10034 Chivasso, Turin, Italy; matteo.cocuzza@infm.polito.it (M.C.); fabrizio.pirri@polito.it (C.F.P.); simone.marasso@polito.it (S.L.M.); 2 Istituto Materiali per Elettronica e Magnetismo, Consiglio Nazionale delle Ricerche, Parco Area delle Scienze, 37a, 43124 Parma, Italy; mosca@imem.cnr.it (R.M.); mvillani@imem.cnr.it (M.V.); zapp@imem.cnr.it (A.Z.); calle@imem.cnr.it (D.C.); 3 Italian Institute of Technology, Center for Sustainable Futures, C.so Trento 21, 10129 Torino, Italy; enis.perrone@iit.it; 4 Department of Information Engineering, University of Parma, Parco Area delle Scienze 181/A, 43124 Parma, Italy; nicola.delmonte@unipr.it

**Keywords:** MEMS, micro-hotplate, microsensor, multiphysics modeling, sensor platform, temperature control, FEM

## Abstract

In the sensors field the active sensing material frequently needs a controlled temperature in order to work properly. In microsystems technology, micro-machined hotplates represent a platform consisting of a thin suspended membrane where the sensing material can be deposited, usually integrating electrical stimuli and temperature readout. The micro-hotplate ensures a series of advantages such as miniaturized size, fast response, high sensitivity, low power consumption and selectivity for chemical sensing. This work compares the coplanar and the buried approach for the micro-hotplate heaters design with the aim to optimize the fabrication process and to propose a guideline for the choice of the suitable design with respect to the applications. In particular, robust Finite Element Method (FEM) models are set up in order to predict the electrical and thermal behavior of the micro-hotplates. The multiphysics approach used for the simulation allows to match as close as possible the actual device to the predictive model: geometries, materials, physics have been carefully linked to the fabricated devices to obtain the best possible accuracy. The materials involved in the fabrication process are accurately selected in order to improve the yield of the process and the performance of the devices. The fabricated micro-hotplates are able to warm the active region up to 400 °C (with a corresponding power consumption equal to 250 mW @ 400 °C) with a uniform temperature distribution in the buried micro-hotplate and a controlled temperature gradient in the coplanar one. A response time of about 70 ms was obtained on the virtual model, which perfectly agrees with the one measured on the fabricated device. Besides morphological, electrical and thermal characterizations, this work includes reliability tests in static and dynamic modes.

## 1. Introduction

Minimization of power consumption is one of the main requirements for sensor technology, especially when targeting the development of portable systems. This is even more crucial when the sensor must work at elevated temperatures, such as metal oxide chemical sensors [1]. Micro Electro-Mechanical System (MEMS) is a high-tech field that combines microelectronics with micromachining technology in order to integrate microcomponents, microsensors, microactuators, signal processing and control circuit [2]. In the common silicon substrate, micro-hotplates generally consist of a thin dielectric membrane suspended over a silicon substrate. In microsystems technology, hotplates are mainly used for sensor applications [3] where the active sensing material is deposited onto the membrane integrated with electrical stimuli and readout [4]. With the development of MEMS technology, that offers a powerful tool to obtain low cost, high efficiency and long-term devices, micro-hotplate has gradually gained wide appreciation for gas sensors [5], infrared emitters, actuators etc. [6]. By employing thin films with good thermal insulation as the membrane structural material, the micro-hotplate presents a series of advantages such as miniaturized size, fast response, high sensitivity and low power consumption [7,8,9,10]. Non-stoichiometric low stress silicon nitride (SiN) grown by Low Pressure Chemical Vapor Deposition (LPCVD) is a very common choice allowing to obtain very flat suspended membranes with high electrical and thermal insulation suitable for the micro-hotplate fabrication as reported by recent literature in this field [11,12,13]. The suspended membrane approach is fundamental to obtain very low heating inertia and high responsive sensors. Heaters and thermometers structures are designed on the SiN membrane to efficiently provide the thermal flux in the sensing region, where thermal driven adsorption or chemical reactions modulate the signal on the sensing elements. Most of the current literature is mainly focused on the development, modelling and optimization of specific and customized micro hot-plates mainly searching straightly uniform heat transfer on the membrane [3,7,9] or, in other cases, exploiting a thermal gradient to obtain multi-sensing array [14,15]. Hence the existing literature usually deals with individual structures of one of the two types above mentioned, eventually with minor and limited variations of the proposed layout (e.g., shape or dimensions of the heater). The main novelty of this paper consists in providing the information and conditions for the parallel development, optimization and modelling of two micro hot-plates fabricated according to two different technological and design paradigms (i.e., buried and coplanar). The two structures are developed in parallel and with analogous characteristics since they share similar designs and dimensions, same materials and fabrication process and same heater aspect ratio. The conditions for the design of the two structures were investigated: technological implementation, critical materials properties characterization (Pt Temperature Coefficient of Resistance and TaOx adhesion layer), construction and simulation of a model with a common and widespread tool (Comsol Multiphysics^®^, Comsol Inc., Burlington, MA, USA), device characterization (thermal, electrical, time response) and long-term reliability test. The parallel treatment and discussion allow to establish a clear and evident comparison and easy to implement design rules for both the structures according to specific and customized needs. Such customized needs may vary from a uniform temperature distribution (generally a more usual requirement satisfied by the buried approach) to a large stable temperature gradient over the suspended membrane (coplanar approach), thus for instance allowing a single heater serving a multi-sensing platform in which several integrated active nanostructures may sense the environment at different operating temperatures. This is known to represent a possible strategy to improve the selectivity for gas sensing applications. The mentioned structures are designed following two different strategies: (i) a coplanar approach where heaters and thermometers are integrated on the same layer as the sensing electrodes, thus resulting on the same geometrical plane; and (ii) a buried approach where they are embedded into an insulating layer, therefore the resistor is placed beneath the sensing electrodes. These two strategies correspond also to different sensing methods: the coplanar approach provides a thermal gradient on the membrane that can be exploited to let the sensor operate at different temperatures at the same instant, while employing a buried approach a very accurate and uniform heat distribution is obtained, thus avoiding any false positive from highly sensitive sensor. From the point of view of fabrication the coplanar approach provides great simplification in terms of process steps and consequent costs with respect to the buried one, since all the processes required to deposit insulation materials on the heater and to electrically separate it from the sensing electrode are not necessary.

In this paper Finite Element Method (FEM) models are set up in order to predict the electrical and thermal behavior of the micro-hotplates following respectively the coplanar and buried approach. The models are based on a multiphysics approach involving electrical, thermal and fluidics aspects. The multiphysics approach has become the leading method to deal with high complex systems that cannot be uniformed on a single physics, and this is particularly evident for MEMS [16,17,18], where electrical, thermal and, sometimes, mechanical behaviors are responsible, at the same time, of the good functionality of the device The geometries, materials and physics are derived from the actual devices to obtain the best possible accuracy. In particular the Pt characteristics were investigated to obtain the experimental Temperature Coefficient of Resistance (TCR) to be included as an input in the models. The two types of devices were fabricated through standard MEMS processes and then characterized. Besides morphological, electrical and thermal characterizations, this work includes reliability tests in static and dynamic modes. 

## 2. Materials and Methods 

### 2.1. Design and FEM Models 

**Figure 1:**
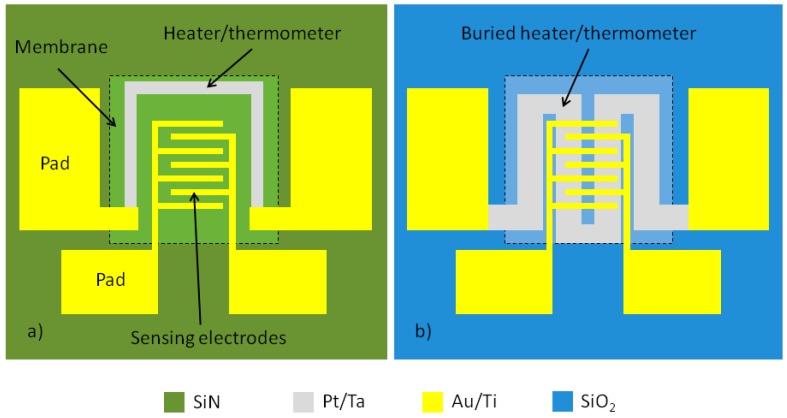
Layout of the micro-hotplate designed according to the coplanar (**a**) and buried (**b**) approaches.

The micro-hotplate sensor is composed by: a thin SiN film membrane, a heater/thermometer Pt resistor, two interdigitated Au sensing electrodes and contact pads (Figure 1). Exploiting this design it is possible to obtain a sensing active region of about 1 mm^2^ (Figure 1). Two different predictive models were carried out by following two designs: a coplanar design with a resistor composed by a Pt thin film patterned around the sensing electrodes (Figure 1a); a buried design where a Pt meandering resistor is positioned on the suspended membrane and buried under an insulating layer beneath the sensing electrodes (Figure 1b). In both of them, the resistor is used for heating the membrane and getting temperature readout through the calibration of the corresponding TCR. The geometrical characteristics of the resistor were tailored in order to endow it with a reasonable room temperature resistance and minimize the heater power consumption. In order to maintain the nominal resistance and to optimize the temperature distribution, the heater has the same length/width aspect ratio in both coplanar and buried micro-hotplate. The readout electrodes were realized with an interdigitated layout as a trade-off between two opposite issues: (i) minimizing the size of the electrodes that represent a high thermal conductive layer placed at the center of the membrane; (ii) maximizing the surface of the electrodes involved in the electrical measurements for sensing.

**Figure 2:**
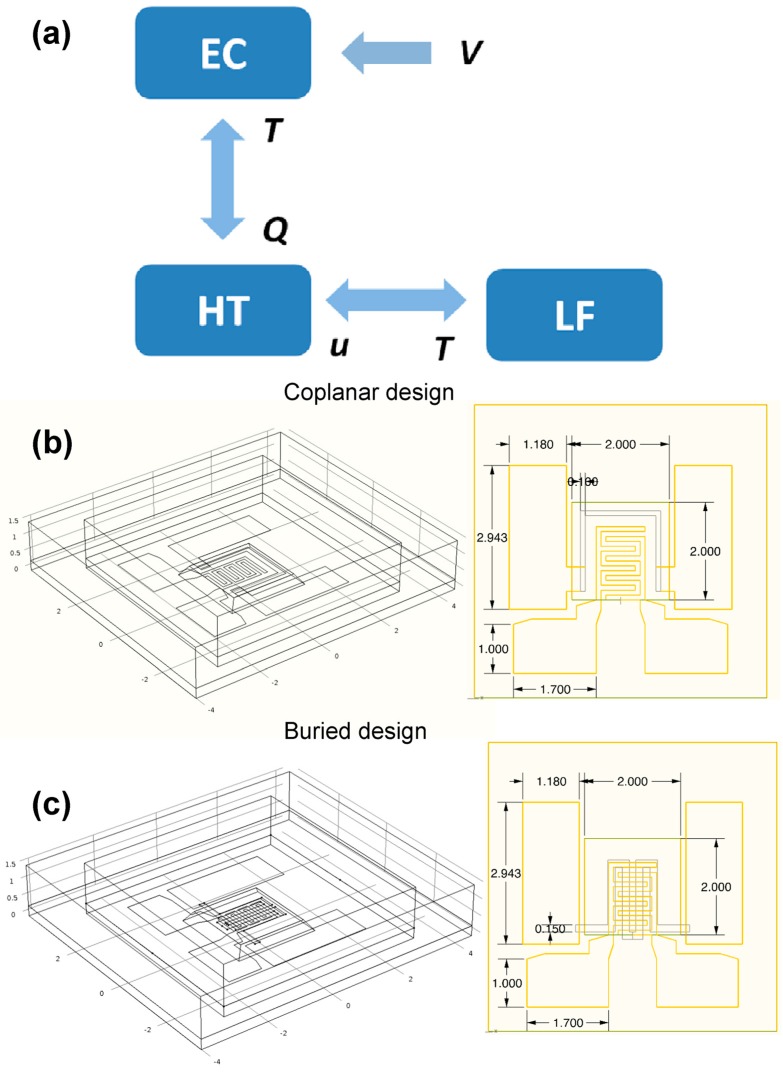
(**a**) Schematic correlation between the equations involved in multiphysics and (**b**) 3D geometries for the coplanar and (**c**) buried models. In the insets the 2D sketches show the exact quotes of the device layout. All the quotes are in mm.

The Finite Element Method (FEM) simulations are carried out in a multiphysics software (COMSOL Multiphysics^®^). The study involves electrical, thermal and fluidics physics that are properly combined to obtain a predictive three-dimensional model. Two different 3D models were generated, which correspond, respectively, to the coplanar and the buried configuration. The device was designed in the simulation environment and an air box of 8 × 8 × 1.5 mm^3^ was placed around the whole silicon chip in order to evaluate the effect of dissipation induced natural air convection (Figure 2). 

The main physical characteristics involved in the model are reported in Table 1 and more details about device geometries are reported in Figure 2.

**Figure 3:**
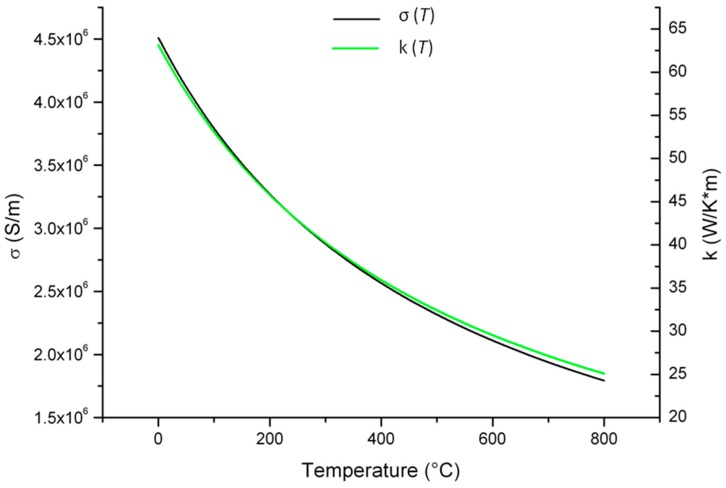
Electrical conductivity σ (*T*) and thermal conductivity k (*T*) of the Pt film used for simulations.

The electrical conductivity of platinum, σ (*T*), were experimentally extrapolated by the Pt film characterization and then inserted as an interpolation curve in the software (Figure 3). In order to obtain a realistic thermal conductivity, the ratio of 1.4 × 10^−5^ WΩ·K^−1^ between the thermal, k (*T*), and σ (*T*) was used [20], thus obtaining a corresponding interpolation curve for k (*T*). 

The equations introduced in FEM model are related to:

(A) the electrical current (EC):
  (1) $$\nabla \cdot J=Q_{j}$$
  (2) J=σE+∂D∂t+Je
  (3) E=−∇V
  (4) $$D=\varepsilon_{0}\varepsilon_{r}E$$
  this equations set represents the dynamic currents equations for a time dependent study [21], **J** is the current density vector, *Q_j_* the charge, **E** the electric field, **D** the electric displacement field, *V* the electric potential, σ is the electrical material conductivity (Table 1), ε_0_ = 8.8541878176 × 10^−12^ F/m is the vacuum permittivity, ε_r_ is the relative material permittivity (Table 1);
(B) the heat transfer (HT):
  (5) ρCp∂T∂t+ρCpu·∇T=∇·(k∇T)+Q
  this equation represents the heat transfer in solids and fluids (air) [22], where C_p_ is the material mass capacitance (Table 1), *T* the temperature, **u** the displacement field, *Q* the heat flux, ρ is the material density (Table 1) and k is the material thermal conductivity (Table 1);
(C) the laminar flow (LF):
  (6) ρair∂u∂t·ρair(u·∇)u=∇·[−pI+μ(∇u·(∇u)T)−23μ(∇·u)I]+F
  (7) ∂u∂t+∇·(ρu)=0
  this equation set is used to simulate the fluids (air) movements forced by convection in the air box [22], where *p* is the pressure, μ the air dynamic viscosity, ρ_air_ the air density, **I** the unit tensor and **F** the external forces. In this case, the default air material properties retrieved by COMSOL Multiphysics^®^ material library were used.

Figure 2a shows the mutual correlation between the equations of the models: the fluidic equations with the thermal ones through the temperature (*T*) and fluid velocity (*u*), while the electrostatic ones with the thermal through the temperature (*T*) and heat flux (*Q*). The aforementioned equations are coupled with the boundary and initial conditions listed in the following: *thin film approach* for all metal layers, which are set with the *electric shielding* condition for EC and in *high conductive layer* condition for HT; one of the heater pad is set at *V* = *V*_drive_, while the other is the ground; all metal layers on the membrane are considered as heat sources; temperature on the bottom surface of the chip is set to 20 °C; on the external surfaces of the model an open boundary condition is set (i.e., fluid normal stress is set equal to *p*_ref_ = 1 atm); fluid is considered as compressible and subjected to a buoyancy volume force (−ρg); at the initial instant (t = 0) fluid pressure is equal to 1 atm; at t = 0 the temperature of the whole device is equal to 20 °C.

### 2.2. Coplanar Fabrication Approach

**Figure 4:**
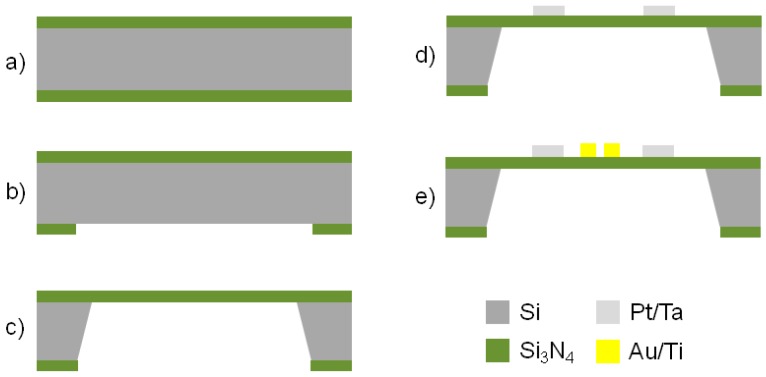
Process flow of the micro-hotplate fabricated according to the coplanar approach. (**a**) Si wafer terminated with low stress SiN (2 μm thick) on both sides; (**b**) RIE of the SiN on the wafer backside; (**c**) KOH wet etching of the Si on the wafer backside; (**d**) patterning of the sputtered Ta (20 nm)/Pt (100 nm) resistor by lift-off; (**e**) patterning of the Ti (20 nm)/Au (200 nm) contact pads and electrodes by lift-off.

The fabrication of the micro-hotplate was performed through a combination of both bulk and surface micromachining. The device was developed starting from a 100 mm diameter, 300 μm thick, double side polished (100) Si wafer, finished with a 2 μm thick coating of SiN on both sides (Figure 4).

Bulk micromachining was employed on the backside of the samples to allow for the realization of the membrane on which the micro-hotplate will be fabricated. These steps include SiN patterning by Reactive Ion Etching (RIE) and silicon anisotropic wet etching in a potassium hydroxide (KOH) aqueous solution. First of all, photolithography on the backside was necessary to pattern the geometries to be etched. For this reason the AZ 9260 positive tone photoresist (MicroChemicals, Ulm, Germany) was spin coated on the wafer backside. The photoresist spin coating process was optimized in order to obtain a thickness of 8.5 μm, which was suitable for the subsequent RIE process to selectively remove the SiN layer from the patterned areas on the wafer backside, thus exposing the underlying Si substrate (Figure 4b). After the photolithographic step, the sample was loaded inside the chamber of a STS 320PC RIE system (Surface Technology Systems, Newport, UK). Sulphur hexafluoride (SF_6_) was used as process gas for the plasma etching. This kind of isotropic SF_6_ etch process has a sufficient selectivity on SiN with respect to a protective resist mask provided with the above mentioned thickness. This allows for a repeatable and relatively cost efficient etching, avoiding the use of a more expensive hard mask protection. The 2 μm SiN layer was etched in about 20 min with a gas pressure of 15 m Torr and an RF power of 150 W. Suspended SiN membranes were obtained by etching 300 μm of bulk Si starting from the opened windows on the backside. The wet anisotropic etching process was accomplished by means of a KOH solution (15 wt % of KOH in water) without using a physical mask to protect the wafer frontside, because KOH solution is completely selective against low stress SiN. The operating temperature of the bath was 85 °C, and the complete removal of the bulk silicon was obtained in about four hours (Figure 4c). 

Next steps involve the deposition and patterning of the resistor (used as heater and thermometer) on the wafer frontside through backside alignment upon the SiN membranes. Surface micromachining starts with the realization of the resistor. The metal depositions described below were performed by using a PVD 75 DC Magnetron Sputtering device (Kurt J. Lesker, Jefferson Hills, PA, USA) at room temperature. The resistor is composed by a Ta (20 nm)/Pt (100 nm) bilayer. The metal patterning was achieved by lift-off employing the image reversal MicroChemicals AZ 5214E photoresist and a dimethyl sulfoxide (DMSO) bath at 60 °C (Figure 4d). The patterned Ta/Pt bilayer was post annealed in a quartz tube furnace at 600 °C for 1 h in air and quenched in order to stabilize the electrical properties. In particular, the resistor was patterned before the heat treatment in order to reduce stresses acting during annealing [23]. Such a stabilization process is essential if the resistor is used in heaters or temperature sensors. In fact, due to progressing agglomeration, the resistive properties of Ta/Pt thin films that operate at high temperatures will drift. However, if the maximum operation temperature is far below a ‘burn-in’ temperature (*T*_burn-in_), the resistive properties are stable for several tens of hours, due to film stress relaxation [23]. Therefore, the heat treatment is expected to raise the *T*_burn-in_ in order to slow the overall degradation. 

Finally, the Ti (20 nm)/Au (200 nm) contact pads and electrodes were fabricated using lift-off (Figure 4e). In particular, the contact pads thickness was tailored in order to allow a functional wire bonding. 

Back-side bulk micromachining was carried out as first step in order to preserve the device active region. Vice versa, releasing the membrane at the end of the fabrication process could introduce a damage of the active region features deriving from the etching process while the toughness of the SiN membrane ensures a very high process yield.

### 2.3. Buried Fabrication Approach

**Figure 5:**
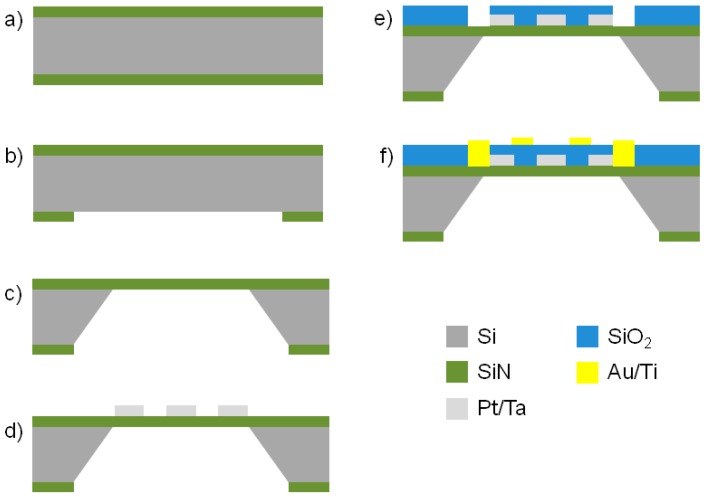
Process flow of the micro-hotplate fabricated according to the buried approach. (**a**) Si wafer terminated with low stress SiN (2 μm thick) on both sides; (**b**) RIE of the SiN on the wafer backside; (**c**) KOH wet etching of the Si on the wafer backside; (**d**) patterning of the sputtered Ta (20 nm)/Pt (100 nm) resistor using aqua regia wet etching; (**e**) patterning of CVD grown SiO_2_ through buffered oxide etch wet etching; (**f**) patterning of the Ti (20 nm)/Au (200 nm) contact pads and electrodes using lift-off.

The fabrication of the micro-hotplate according to the buried approach has some shared steps with respect to the fabrication of a coplanar micro-hotplate. The device was developed starting from a 100 mm diameter, 300 μm thick, double side polished (100) Si wafer, finished with a 2 μm thick coating of non-stoichiometric low stress silicon nitride (SiN) on both sides (Figure 5a). The membrane was realized through bulk micromachining: SiN patterning by Reactive Ion Etching (Figure 5b) and silicon anisotropic wet etching in a KOH aqueous solution (Figure 5c). Surface micromachining involves the deposition and patterning of the resistor on the wafer frontside through backside alignment upon the SiN membranes and subsequently the deposition and patterning of the electrodes and contact pads. The resistor was composed by a room temperature sputtered Ta (20 nm)/Pt (100 nm) bilayer patterned by wet etching through aqua regia (3 HCl:1 HNO_3_) at 70 °C (Figure 5d). As described, the patterned resistor was post-treated at 600 °C in order to stabilize the resistive properties. 

SiO_2_ was chosen to bury the resistor and ensure the electrical insulation with respect to the measuring electrodes. Indeed, an 800 nm thick layer of SiO_2_ was grown through a Plasma-Enhanced Chemical Vapor Deposition (PECVD) system (Elettrorava S.p.A., Venaria Reale (TO), Italy, 15 W, 13.56 MHz, 500 °C, 450 mTorr, 4 sccm SiH_4_, 70 sccm CO_2_, 100 sccm H_2_) and patterned by wet etching using a Buffered Oxide Etch (B.O.E.) solution and a positive tone photoresist as masking layer (HPR 504 photoresist, Fujifilm, Valhalla, NY, USA; Figure 5e). The same etching allowed to expose the electrical contacts of the buried resistor. The choice of SiO_2_ as insulating material was mainly due to its selectivity with respect to the low stress SiN membrane, which acts as a perfect etch-stop layer avoiding under etching that could damage the electrical continuity of the metallization. The SiO_2_ thickness was properly tailored to reach an optimal compromise between the maximum electrical insulation, which should be orders of magnitude higher with respect to the nanostructured sensitive layer resistance, and the minimum gap between the resistor and sensitive layer, which should go up to 400 °C to properly work.

Finally, Ti (20 nm)/Au (200 nm) contact pads and electrodes were fabricated using a lift-off step (Figure 5f) according to the process used in the coplanar micro-hotplate.

### 2.4. Device Characterization 

After the most critical process steps a deep morphological and materials analysis was carried out. In particular the Pt film resistor has been characterized after the thermal annealing performed at 600 °C using Field Emission Scanning Electron Microscopy (FESEM, SUPRA 40, Zeiss, Oberkochen, Germany) and X-ray Photoelectron Spectroscopy (XPS, Versa probe 5000, PHI, Chanhassen, MN, USA). The TCR was obtained in a climatic chamber (Climatic System TY80 Angelantoni, Località Cimacolle (PG), Italy) using an model 34970A data logger (Agilent, Santa Clara, CA, USA, equipped with the 34901A board) for the temperature (20–70 °C) and resistance acquisitions. The long-term stability and the response time of the devices were collected with the same data logger by applying a periodic step driving voltage of *V*_drive_ = 5.7 V with a period of 8 s and a duty cycle of 50%. In particular, a 4200 SCS Semiconductor Characterization System (Keithley, Cleveland, OH, USA) was employed for the response time evaluation, while the Agilent data logger 34970A was used for the long term stability measurements.

Thermal imaging was carried out by using an A325 camera (Flir, Wilsonville, OR, USA) equipped by a Flir close-up lens 2 × (50 μm) to increase image resolution.

## 3. Results and Discussion

In micromechanics SiN is very often used as a structural material. LPCVD SiN is particularly relevant because it is characterized by a high fracture toughness and a good thermal shock resistance that allow the membrane to withstand high working temperature. Moreover, its low thermal conductivity involves a low heater power consumption.

The materials generally used as Resistance Temperature Detectors (RTDs) are platinum, copper, nickel and nickel iron alloys. However, Pt is the primary choice for most industrial, commercial, laboratory and other critical temperature measurements because of its resistance to oxidation, best accuracy and chemical and thermal stability also at high temperatures [23,24,25]. Similarly intrinsic properties like high conductivity, chemical and thermal stability, and suitability for wire bonding make Au the most favourable material in order to realize sensing electrodes and contact pads in a single fabrication step.

According to the described process conditions, Ta was used as adhesion layer because it has proven to be better than other materials like Ti. In fact, the Ta layer is able to withstand annealing in an oxidizing atmosphere at 600 °C. This is in contrast to the optimal solution on the SiO_2_ barrier layer, where the TiO_2_/Ti is by far the best choice [26]. The evidence of the deterioration of the Ti/Pt bilayer in oxidizing environments at high temperatures is fully explained. Annealing in an oxidizing atmosphere causes diffusion of O_2_ through the Pt columnar grain boundaries, rapid oxidation of the underlying Ti layers and its migration into Pt. These phenomena generate significant problems in terms of loss of adhesion and degradation of Pt resistive properties [27]. Such adverse reactions could not be prevented despite the deposition of Pt at high temperatures for achieving effective densification in the Pt layers. Improved stability is usually obtained by stabilizing the interface chemistry by introducing a well reacted oxide layer at the interface between Pt and the Si-based substrate. In this view, Ta plays an important role in the diffusion phenomena because during the heat treatment it forms a stable oxide increasing its thickness [23,28]. In particular, Ta oxidizes because oxygen diffuses through the Pt grain boundaries and, in contrast to the case of TiO_x_, the underlying relaxation mechanism is a recrystallization process rather than a diffusion one [23]. As Ta naturally oxidizes, the introduction of the oxide barrier is not performed through a deposition process, resulting in a simplification of the micro-hotplate fabrication process. Such recrystallization processes may affect the device functionality also through the formation of hillocks that increment the roughness of Pt surface [23,29,30,31,32]. 

**Figure 6:**
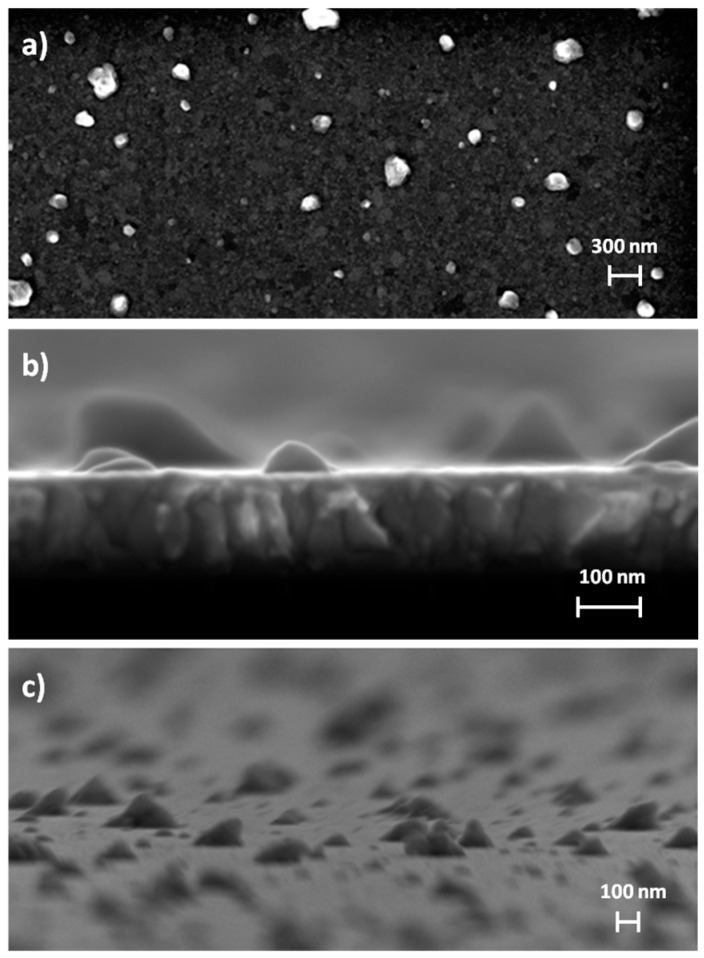
FESEM images of the resistor: (**a**) top view; (**b**) cross-section view and (**c**) tilted view.

**Figure 7:**
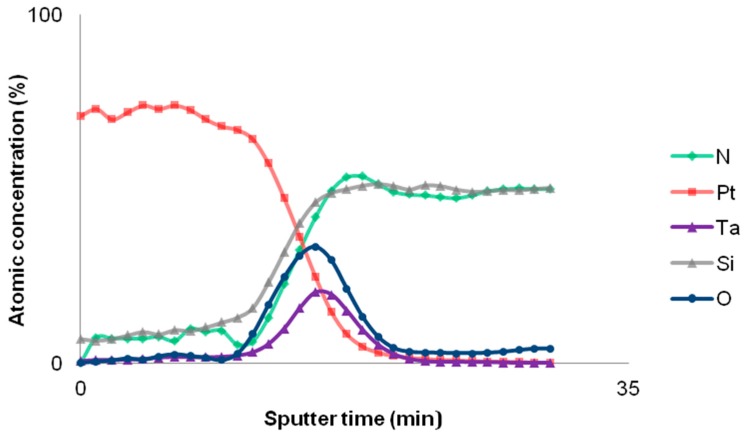
Depth profile analysis of the resistor performed by X-ray Photoelectron Spectroscopy (XPS). The atomic concentrations are reported as a function of sputter time.

The morphological analysis performed with a Field Emission Scanning Electron Microscope confirms the expectations of the literature survey: the surface of the resistor is full of hillocks with different sizes. In the top view reported in Figure 6a, hillocks appear as white islands that seem out of focus with respect to the Pt surface. Actually, hillocks are raised grains characterized by multiple dimensions and a different crystalline orientation with respect to the orientation of thin films [32]. Figure 6b shows a cross-section view of the resistor in which the Pt columnar grain structure is remarkable as well as the presence of hillocks on the top. Figure 6c is a tilted view that attests the distribution of hillocks on the whole Pt surface. Regarding the buried approach, heat treatment plays a key role in the evaluation of insulating layer thickness because of its correlation with the formation of hillocks which increase in number as well as in size with temperature, inducing a significant surface roughness [32]. As a consequence, the SiO_2_ thickness was tailored in order to properly embed the resistor and get the electrical isolation. The depth profile analysis performed by XPS is able to provide information about the atomic composition of the resistor. The atomic concentration of the resistor is reported as a function of the sputter time in Figure 7. The point of interest is situated at the interface between the resistor and the SiN of the substrate, where percentages of Ta and O_2_ are clearly detected by XPS. Such an evidence fully agrees with the aforementioned discussion about the oxidation of Ta adhesion layer due to the heat treatment. As a consequence the resistor, initially composed by a room temperature sputtered Ta/Pt bilayer, finally involves a thin layer of tantalum oxide. As TaO_x_ is an insulator, the current injected in the resistor flows exclusively through the Pt layer which accordingly requires a proper design in order to tailor the resistive properties. 

The aim of the resistor is to heat the membrane and contextually measure the temperature reached on the membrane exploiting the Joule effect. Knowing the behavior of the electrical resistance as a function of the temperature is fundamental in order to properly use the resistor as heater and thermometer. In this view, the TCR describes the relative change of a resistance associated with a temperature change and is defined by Equation (7) in which α has the dimension of an inverse temperature (°C^−1^ or K^−1^). If the temperature coefficient itself does not vary too much with temperature, a linear approximation (Equations (7) and (8)) can be used to determine the value of the resistance *R* at a temperature *T*, using the Callendar–Van Dusen equation [33], given its value *R*_0_ at a reference temperature *T*_0_ (usually room temperature):

(8) dRR=αdT

(9) $$R\left(T\right)=R\left(T_{0}\right)\left(1+\alpha ∆T\right)$$

**Figure 8:**
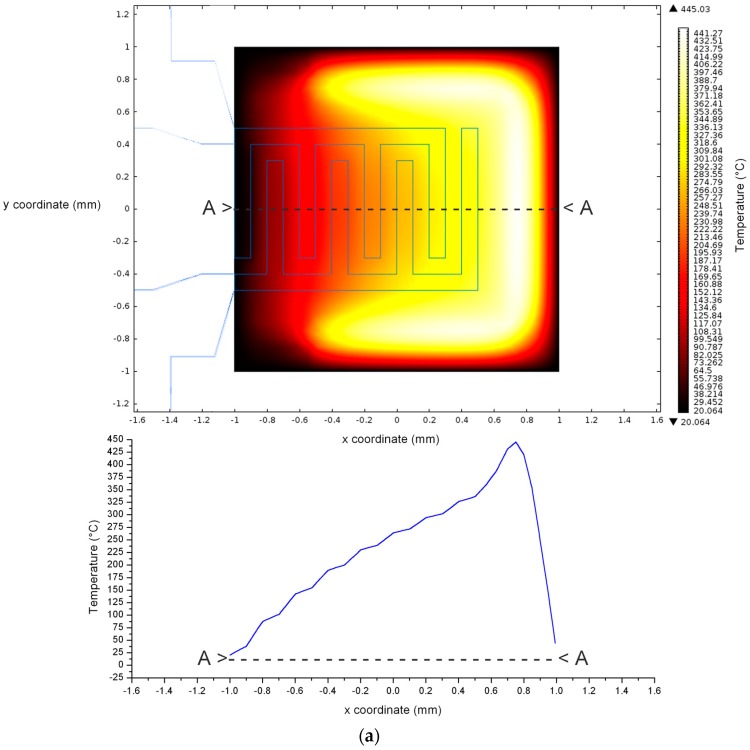

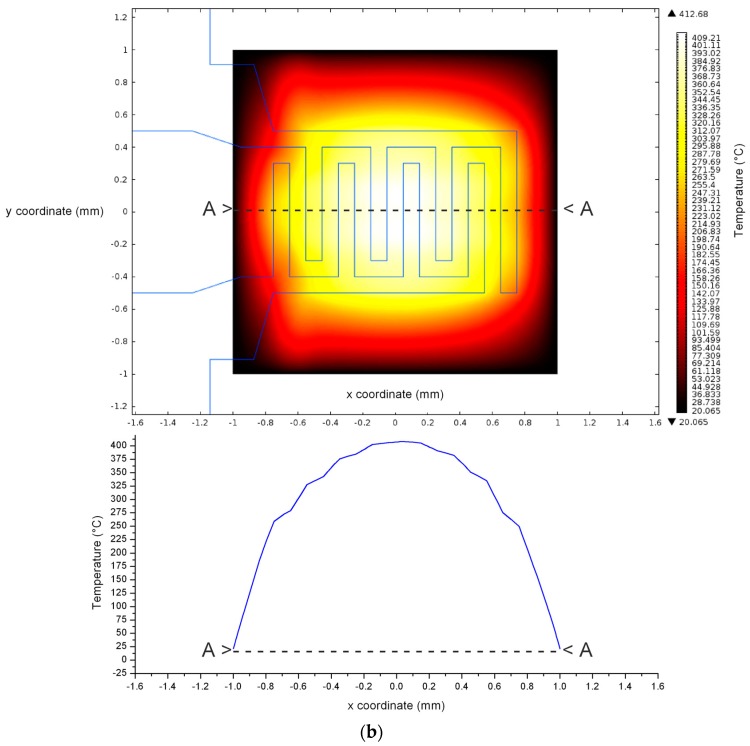
Temperature distribution on SiN membrane obtained by FEM models with 5.7 V applied to the thin film heater. Both the surface view and the section temperature profiles were reported for: (**a**) coplanar heater design; (**b**) buried heater design.

**Figure 9:**
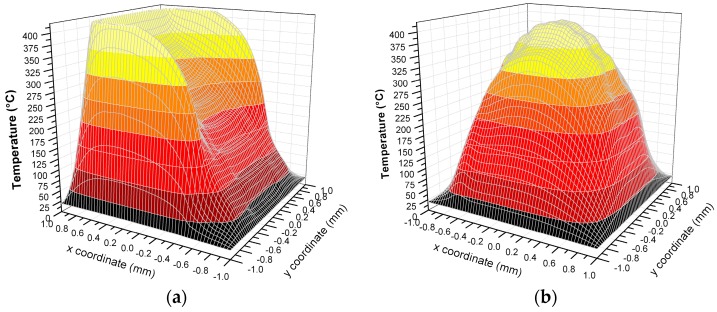
3D plot of the temperature distribution on SiN membrane obtained by FEM models with 5.7 V applied to the thin film heaters for: (**a**) coplanar heater design; (**b**) buried heater design.

Therefore, evaluating the TCR of the resistor is mandatory in order to univocally associate the resistance value to the real temperature reached on the resistor. It is known that the resistive properties of a bulk material are quite different with respect to the properties of a thin film. Thus, the TCR of TaO_x_/Pt bilayer, that is far different from that of Pt bulk (3.92 × 10^−3^ °C^−1^) [23], has to be experimentally evaluated. Measuring the variation of resistance as a function of temperature over the range 20–70 °C (59.75 Ω @ 20 °C and 65.15 Ω @ 70 °C) and using the Equations (8) and (9), the average TCR for the fabricated resistor is equal to 1.81 × 10^−3^ °C^−1^. Knowing the TCR allows one to tailor the supplying voltage and the related power consumption necessary to reach a temperature set point. Using this experimental value it is possible to evaluate the temperature distribution on the SiN membrane (Figure 8 and Figure 9) by the FEM models. The plots reported on Figure 8 and Figure 9 demonstrate that the predictive models are fundamental tools to tailor the design of a sensor, and in general of a microdevice, since they provide information impossible to be extracted from a simple characterization. The hypothesis beyond this assertion is that data from experimental analysis should match the model results, but, of course, an improvement of the same is always possible by changing parameters or introducing correction factors. An evaluation of the SiO_2_ film on the buried design was also carried out, but there is no significant contribution from this layer to the device performance or temperature distribution since a difference of the order of +0.1 °C was found on the same probe point on the bottom with respect to the top of the SiO_2_ surface.

The virtual models results demonstrate that the working temperature (400 °C) of the sensor [11] is reached with 5.7 V applied to the thin film heater. Figure 8a shows a linear temperature gradient formation on the membrane that can vary from 400 °C in the region next to the Pt resistor to about 150 °C close to the pads of the sensing electrodes. In order to quantify the linear gradient, the variation of temperature in the sensing region along the A-A section was evaluated: a linear coefficient between the temperature and the x coordinate of 0.2 °C/μm was found. This means that a linear variation from 25 °C to 400 °C that increases of 0.2 °C every micron, can be stably maintained along the A-A section (Figure 8a). As expected, the temperature distribution of the buried design model is actually uniform especially in correspondence of the sensing region, where the set value of temperature can be spatially maintained with a maximum deviation of 7% with respect to the maximum temperature reached at the center (Figure 8b). Moreover if an area of 200 × 200 μm^2^ around the membrane center is considered, the temperature maximum deviation with respect to the maximum decreases to 2% (Figure 8b).

**Figure 10:**
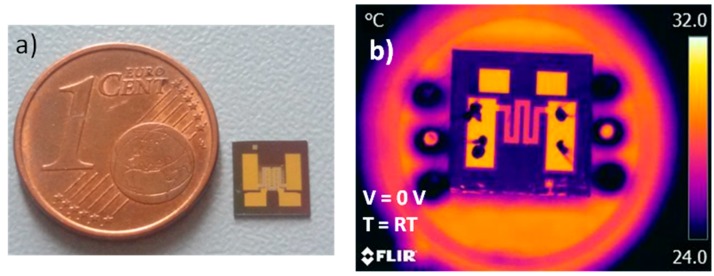
(**a**) Buried micro-hotplate die; (**b**) infrared thermography of the micro-hotplate bonded in TO-8 package.

**Figure 11:**
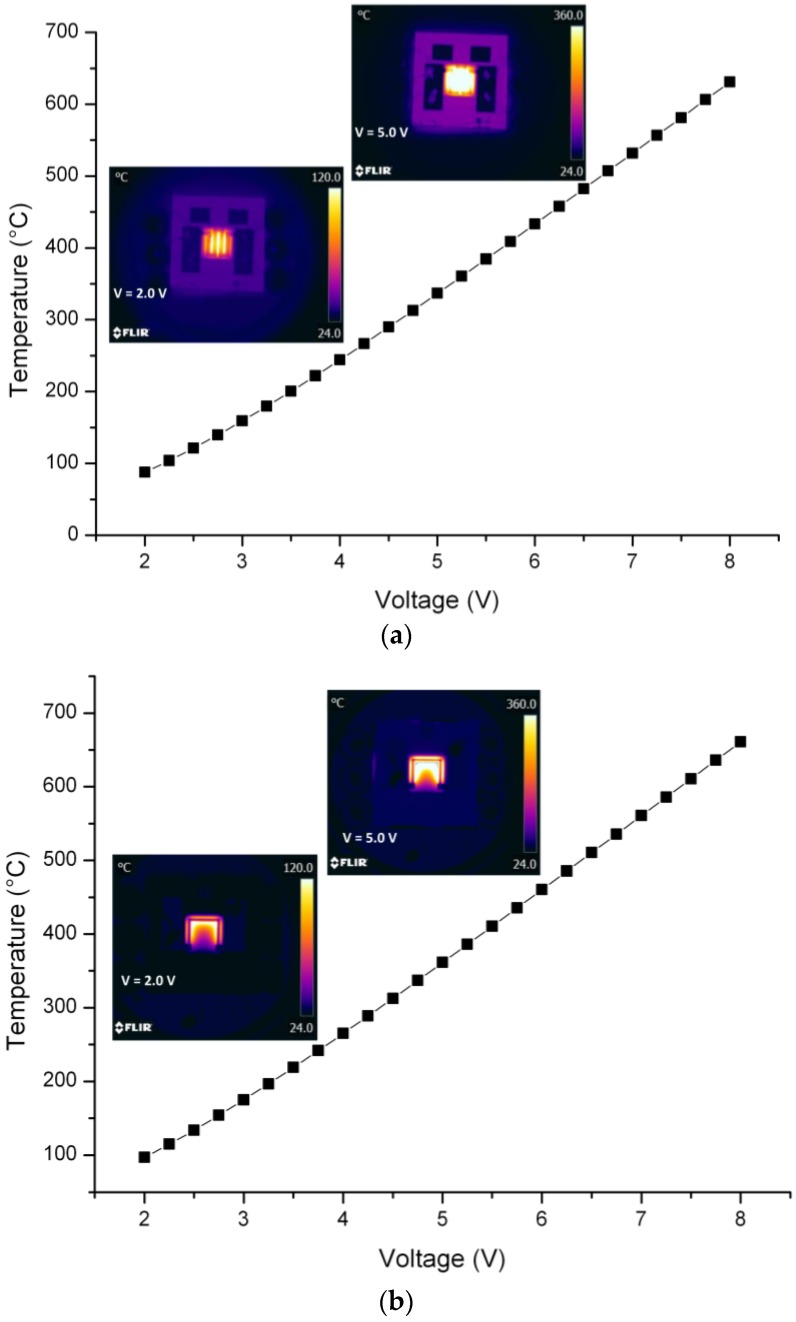
Comparison between the temperature variation of the resistor as a function of the input voltage as from the model and thermography characterization on the device for the buried (**a**) and coplanar (**b**) design.

The micro-hotplate die (Figure 10a), having a square shape sized 6 × 6 mm^2^, is compatible with the standard TO-8 package. The wire bonding is realized through gold ball bonding and the die remains floating and well anchored to the electrical connections. Such a set-up is used to carry out the infrared thermography analysis. The actual device characterization confirms the simulated behavior: (i) the device reaches 400 °C with 5.7 V applied to the thin film heater; (ii) the temperature distribution on SiN membrane is corroborates by the thermography analysis on the sensor as reported in the comparative plots in Figure 11. Using this results it is possible to predict very accurately the working temperature on the SiN membrane and then use this data to calibrate the final device selectivity [11].

Using the simulated behavior of the micro-hotplates it is possible to obtain the characteristic temperature curve of the resistors at different voltages (Figure 11). The linear trend is a typical property of the Pt film, but this assertion is verified only if a proper thermal annealing at a sufficient higher temperature is performed. For this reason the devices have been thermally treated at 600 °C during the fabrication process as already described. In the coplanar design the U-Shape thermography replicates the design of the Pt resistor very well. This suggests that the temperature gradient can be geometrically designed as a function of the resistor geometry. Therefore, the predictive model can be easily employed to check the geometry variation influence on the temperature gradient. The meandering design of the buried resistor ensures the desired temperature uniformity in the sensing area, thus providing the ideal solution for every micro-hotplate based sensor in which the active material is very sensitive to the temperature variation and needs a high heat flux to work properly. 

**Figure 12:**
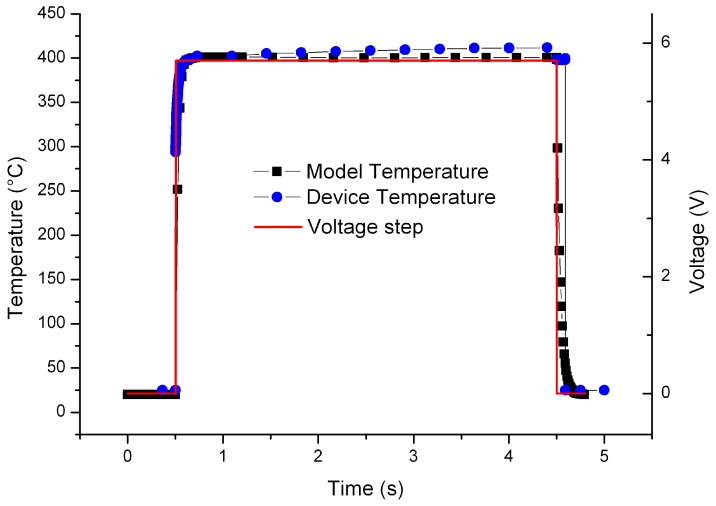
Response time to a step voltage input for the buried design: comparison between the model and the actual device.

The response time of the models and the actual device have been investigated in order to compare the ramp up velocity (Figure 12). The response time was defined as the time to reach the plateau temperature within an error of ±5% with respect to the plateau nominal temperature. In the case of the buried design, for the selected voltage of 5.7 V, it was expected a temperature plateau of 407 °C on the resistor. The simulations results indicate a delay of the order of tens of milliseconds between the driving voltage ramp up and temperature plateau and then a resulting response time of 70 ms. For the actual device a response time of 75 ms was obtained. These results confirm the model prediction and can be used to obtain a very fast microhotplate based sensor design, in particular, to evaluate the ideal response time of the sensor, to tune the driving voltage waveform, to define the plateau temperature at a specific voltage, etc. The same result in terms of time response was obtained on the coplanar design. A typical temperature drift due to the rapid rising up of the voltage is shown (Figure 12) for the actual device.

**Figure 13:**
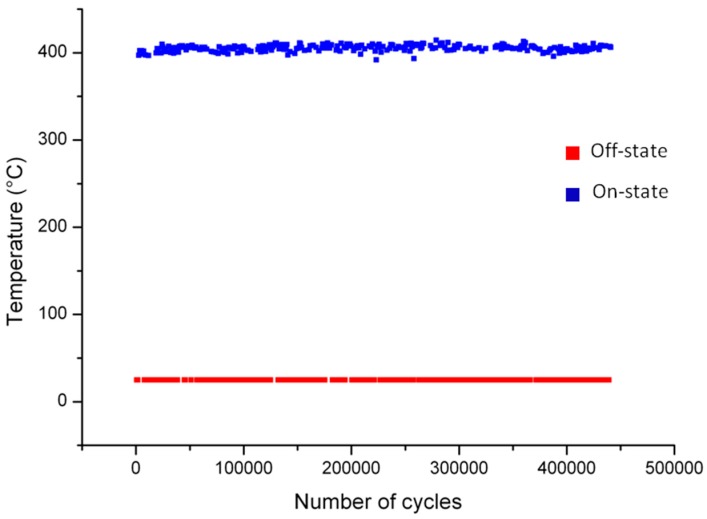
Long term on/off cycling test on the fabricated devices.

The actual resistor is able to warm up to 400 °C with a power consumption of about 250 mW. Under these conditions, the properties of the resistor have been tested for a period of three weeks during which no appreciable changes have been observed (Figure 13) already used This means that the Pt film has a very high stability in terms of conductivity and, even if directly exposed to a not clean environment without any package, it does not alter its behavior. Again it depends from the selection of the Ta as adhesive layer for the Pt film and the thermal treatment at 600 °C, 200 °C above the working temperature, which avoids any resistor degradation. This confirms that this kind of devices is suitable to develop efficient and robust sensors.

## 4. Conclusions

Micromachined hotplates are conceived as platforms for sensing applications. A FEM method based on a multiphysics approach was implemented in order to predict the electrical and thermal behaviour of the micro-hotplates. The platform was designed taking into account two different configurations, namely the coplanar and buried ones. The fabrication specifications adopted, based on a careful analysis of the literature and corroborated by morphological and electrical characterizations, led to an optimized fabrication process. The fabricated micro-hotplates are able to warm the active sensing region of the membrane with a uniform temperature distribution in the buried micro-hotplate and a controlled temperature gradient in the coplanar one. Moreover a response time of 70 ms to reach the working temperature of about 400 °C was estimated by the virtual model and confirmed on the actual device. In-fact, the experimental data prove the predicting model validity opening its employment also in devices with a more sophisticated layout. The electrical tests in static and dynamic modes verify the device reliability and the efficiency of the fabrication process. This study demonstrates that fundamental information of a micro-hotplate, as for example the temperature distribution, can be easily deducted and plotted exploiting the virtual model, therefore re-design or performance check can be performed on the model without facing expensive device fabrication processes. More than this, also the characterization costs can decrease as demonstrated by the evaluation of the response time.

## Figures and Tables

**Table 1:** Physical properties of the materials involved.

Property (Units)	Materials			
	Si	SiN	Au	Pt
Density ρ (kg/m^3^)	2330	3100	19,300	21,450
Rel. permittivity ε_r_	11.7	9.7	1	1
El. Conductivity σ (S/m)	0.1	10^−5^	2.5 × 10^7^	σ (*T*)
Specific Heat C_p_ (J/(kg·K))	700	700	129	133
Th. Conductivity k (W/(m·K))	130	6 [19]	160	k (*T*)
